# Editorial: The role of alcohol in modifying behavior

**DOI:** 10.3389/fnbeh.2023.1175405

**Published:** 2023-03-30

**Authors:** Jamie Peters, Luigia Trabace, Giuseppe Di Giovanni

**Affiliations:** ^1^Department of Anesthesiology, University of Colorado Anschutz Medical Campus, Aurora, CO, United States; ^2^Department of Pharmacology, University of Colorado Anschutz Medical Campus, Aurora, CO, United States; ^3^Department of Clinical and Experimental Medicine, University of Foggia, Foggia, Italy; ^4^Department of Physiology and Biochemistry, Faculty of Medicine and Surgery, University of Malta, Msida, Malta; ^5^Neuroscience Division, School of Biosciences, Cardiff University, Cardiff, United Kingdom

**Keywords:** alcohol, behavior, stress, adolescence, sex

This special topic presents experimental work on the effects of alcohol (ethanol) on the brain, and how these effects impact behavior across multiple domains. The World Health Organization (WHO) estimates that 2.3 billion people regularly consume alcohol, making alcohol one of the most widely used drugs in human society (WHO, 2022). Alcohol consumption has both acute and long-term effects on behavior. Whereas most of the acute effects are rewarding, if higher doses are consumed, negative effects including motor and cognitive impairment can occur and can be lasting. Although recreational use of alcohol can enhance sociability, excessive repeated alcohol use can lead to alcohol use disorder (AUD) and physical dependence associated with a dangerous withdrawal syndrome, such as delirium tremens. Substance use disorders are characterized by frequent comorbidity with the use of other substances, and alcohol is commonly co-used with other substances, including psychostimulants like cocaine, as well as opioids (Bobashev et al., 2018; Cicero et al., 2020). Comorbid use of substances increases the risk of adverse outcomes and relapse (Wang et al., 2017), and this complexity of the human condition requires the use of preclinical animal models to tease apart the complex effects of alcohol on behavior (Crummy et al., 2020).

Animal models play a crucial role in understanding the effects of alcohol on the brain and behavior (Mineur et al., 2022; Valyear et al., 2023). Studies have shown that a range of brain structures are involved in alcohol use including the amygdala, nucleus accumbens, and insula. Targeted stimulation and suppression of these areas of the brain is able to alter alcohol consumption. For instance, Haaranen et al. used a chemogenetic approach to alter neuronal activity in these individual brain regions, and in the specific insula outputs to the nucleus accumbens and basolateral and central subregions of the amygdala, to determine the functional role of this network on alcohol consumption in alcohol preferring Alko Alcohol (AA) rats. This type of sophisticated circuit-level analyses is necessary to understand how neural networks function to control alcohol consumption, in order to design targeted treatment strategies aimed at altering network function. The previous study found that activating the insula projections to amygdala or nucleus accumbens increased alcohol consumption, consistent with prior work demonstrating the insula is a critical driver of alcohol relapse (Campbell et al., 2019). Emerging potential new medications for treating AUD like Glucagon-Like Peptide 1 (GLP-1) may work in part by decreasing cue-associated craving-related increases in insula activity, as systematically reviewed by Eren-Yazicioglu et al. in this special edition.

Alcohol use can be triggered by numerous factors, and stress is one of the most potent triggers for craving and relapse (Wemm et al., 2019). Interestingly, Deal et al. found that both social and non-social stressors enhance the release of catecholamines in the basolateral amygdala, and acute alcohol blunts this stress response, perhaps providing a brain-based rationale for the self-medication hypothesis (Ayer et al., 2010). This adds to a growing body of literature implicating the amygdala as an important brain site by which stress can alter alcohol seeking and use (Mineur et al., 2022). Furthermore, while the health benefits of daily exercise cannot be denied, the study by Buhr et al. suggests that it does not alter alcohol's effects on serotonin and dopamine-related turnover in the striatum and brain stem. However, alcohol drinking altered neurochemical correlates of exercise in the hypothalamus, a key component of brain networks responsible for maintaining physiological homeostasis. As demonstrated in the study by Starski et al., repeated and prolonged alcohol use can lead to allostasis and further exacerbate behavioral engagement with alcohol. Furthermore, the behavioral and brain response to stress is sexually dimorphic, and the brain response to stress and drug cues predicts subsequent relapse (Smith et al., 2023).

Genetic factors, as well as age and sex, can influence alcohol use and behavioral phenotypes associated with alcohol use. Alcohol drinking often begins in adolescence (Abela et al., 2023), and the study by Corongiu et al. demonstrates that adolescents typically drink more alcohol than adults, but that this precise phenotype interacts with genetic background. Moreover, sex can influence alcohol use and behavior, with AUD being diagnosed more often in men than women. In line with this, Bryant et al. found that male mice were more sensitive to the motivating effects of alcohol, and Landin and Chandler report that male rats exposed to alcohol during adolescence were more prone to have greater behavioral responses to threat in adulthood, although females were already predisposed to this phenotype, regardless of alcohol history. To make matters more complex, the neurobiological hallmarks of adolescent alcohol exposure may be sexually dimorphic. For example, Asarch et al. found that in male rats, mesolimbic dopamine peaks during adolescence then declines and stabilizes in adulthood, but adolescent alcohol exposure prolongs the elevated dopamine levels into adulthood, an “arrested development” phenotype not observed in female rats, whose dopamine levels are stable throughout adolescence and adulthood. On a more positive note, Rodd et al. report that negative allosteric modulators of the nicotinic α7 receptors may hold promise as prophylactics against the deleterious effects of binge alcohol use during adolescence.

Overall, the contributions to this special topic have broadened our understanding of how, where, and when alcohol acts in the brain to promote continued alcohol use, which in some individuals can lead to full blown AUD. The extensive comorbid use of alcohol with other drugs is also of growing concern and calls for novel preclinical models of polydrug use to determine the neurobiological consequences of comorbid alcohol use with other substances and to effectively screen emerging therapeutics. Continued research in this area is needed in order to develop novel treatment interventions, including prophylactics, medications, and natural remedies.

## Author contributions

JP wrote the original editorial draft. All authors edited and revised the editorial.
